# Supplementary material for: Features of the Preparation and Luminescence of Langmuir-Blodgett Films Based on the Tb(III) Complex with 3-Methyl-1-phenyl-4-stearoylpyrazol-5-one and 2,2′-Bipyridine
Source: Materials (Basel). 2022 Jan 31;15(3):1127. doi: 10.3390/ma15031127 (PMC8840368; doi:10.3390/ma15031127)
Supplement: Supplementary file 1 [file materials-15-01127-s001.zip › materials-1539589-supplementary.pdf]

materials

Supplementary Materials

Features of the Preparation and Luminescence of Langmuir-Blodgett Films Based on the Tb(III) Complex with 3-Methyl-1-Phenyl-4-Stearoylpyrazol-5-One and 2,2′-Bipyridine

Julia Devterova [1], Kirill Kirillov [2], Anton Nikolaev [1], Michail Sokolov [2], Victor Shul'gin [3], Alexey Gusev [3], Victor Panyushkin [1] and Wolfgang Linert [4,*]

1 Department of Chemistry and High Technology, Kuban State University, 350040 Krasnodar, Russia; devterova8julia@gmail.com (J.D.); map_kompass@mail.ru (A.N.); panyushkin@chem.kubsu.ru (V.P.)
2 Department of Physics and Technics, Kuban State University, 350040 Krasnodar, Russia; kirillka.kir@bk.ru (K.K.); sokolovme@mail.ru (M.S.)
3 Institute of Biochemical Technology, Ecology and Pharmacy, V.I. Vernadsky Crimean Federal University, 295007 Simferopol, Russia; shulvic@gmail.com (V.S.); galex0330@gmail.com (A.G.)
4 Institute of Applied Synthetic Chemistry, Vienna University of Technology, A-1060 Vienna, Austria
* Correspondence: wolfgang.linert@tuwien.ac.at; Tel.: +43-(1)-58801-163613

Figures

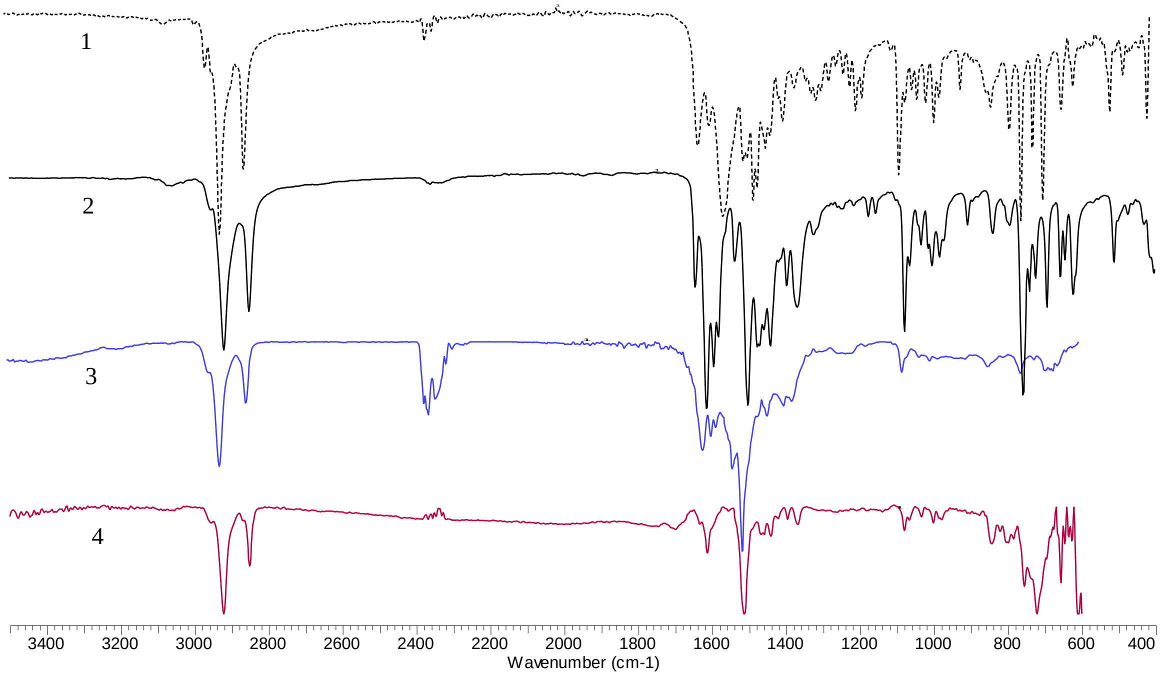

Figure S1. IR spectra of 1) HL (powder), 2) TbL_3·bipy (powder), 3) 10-layer TbL_3·bipy-based LBF obtained on subphase with $C(Tb^{3+}) = 5{\cdot}10^{-4}$ M, 4) 10-layer TbL_3·bipy-based LBF obtained on subphase without terbium ions, $C(Tb^{3+}) = 0.0$ M.

© 2022 by the authors. Submitted for possible open access publication under the terms and conditions of the Creative Commons Attribution (CC BY) license (http://creativecommons.org/licenses/by/4.0/).
